# (Re)conceptualizing movement behavior in sport as a problem-solving activity

**DOI:** 10.3389/fspor.2023.1130131

**Published:** 2023-05-26

**Authors:** Shawn Myszka, Tyler Yearby, Keith Davids

**Affiliations:** ^1^ Emergence, Minneapolis, MN, United States; ^2^ School of Natural, Social and Sport Sciences, University of Gloucestershire, Gloucester, United Kingdom; ^3^ Sport & Physical Activity Research Centre, Sheffield Hallam University, Sheffield, United Kingdom

**Keywords:** movement behavior, problem-solving, ecological dynamics, dexterity, perception-action coupling, affordances (ecological psychology)

## Abstract

The use of the term *problem-solving* in relation to movement behavior is an often-broached topic within kinesiology. Here we present a clear rationale for the concept of problem-solving, specifically pertaining to the skilled organization of movement behaviors in sport performance, and the respective processes that underpin it, conceptualized within an *ecological dynamics* framework. The movement behavior that emerges in sport can be viewed as a problem-solving activity for the athlete, where *integrated movement solutions* are underpinned by intertwined processes of perception, cognition, and action. This *movement problem-solving process* becomes functionally aligned with sport performance challenges through a tight coupling to relevant information sources in the environment, which specify affordances offered to the athlete. This ecological perspective can shape our lens on how movements are coordinated and controlled in the context of sport, influencing practical approaches utilized towards facilitating dexterity of athletes. These ideas imply how coaches could set *alive movement problems* for athletes to solve within practice environments, where they would be required to continuously (re)organize movement system degrees of freedom in relation to dynamic and emergent opportunities, across diverse, complex problems. Through these experiences, athletes could become attuned, intentional, and adaptable, capable of (re)organizing a behavioral fit to performance problems in context—essentially allowing them *to become one with* the movement problem.

## Introduction

With only seconds left to play in the championship game and her team currently behind by a single point, a basketball point guard advances the ball down the court, dribbling while she weaves seamlessly through the traffic of others. She continuously remains ready to pass the ball in a moment's notice as she scans the landscape for an open teammate, who may be afforded a scoring opportunity. A heavyweight boxer stands just a few feet from an opponent who has sincerely bad intentions. He moves in and out of “the pocket”, attempting to stay in range just long enough to detect an opening based on his opponent's behaviors, throw a number of punches in combination, and quickly back out of this danger zone to a distance where there is less risk. A running back takes a handoff from the quarterback, and within fractions of a second, he is met in the gap by an aggressively pursuing linebacker who is just a couple of yards from him. This culminates in the need for the back to appropriately perceive the situation, make a rapid but accurate decision, and carry out a movement strategy where he escapes from the tackle attempt of the defender. After solving the first problem, the back will need to immediately solve another emerging problem in the performance landscape.

It is clear that complex movement problems exist everywhere within sport at all levels, and they continuously present both challenges and opportunities, of varying relative intensities, to athletes of all demographics. As Verkhoshansky and Siff (1) stated, “sport then becomes a problem-solving activity in which movements are used to produce the necessary solutions.” Skillful athletes are not always bigger, faster, and stronger than their peers, but they are often those able to coordinate their movement responses in solving a wider variety of problems, often within challenging and unpredictable contexts, while performing under the constraints of immense pressure, fatigue, and other potential perturbations.

The use of the term *problem-solving* in relation to movement behavior is an often-broached topic within kinesiology. However, a clear definition of this concept of problem-solving, especially as it pertains to skilled movement behavior in sport, and the respective processes which may underpin it, is worth articulating within an ecological dynamics framework. Thus, the purpose of this conceptual analysis is to adopt an ecological perspective in evaluating theory and evidence as to how athletes may solve movement problems in sport. Viewing movement behavior (i.e., the coordination, control and regulation of actions) in sport as a problem-solving activity can also bring important practical implications. An ecological conceptualization emphasizes the contexts of performance and could influence the nature of practice activities and designs used by practitioners, with the goal of facilitating dexterity (2) within athletes. While the primary target audience of this conceptual analysis is coaching practitioners and skill acquisition specialists working within sports, we hope that the ideas help shape how others (e.g., those in research) conceptualize and study movement behavior in sports.

Various conceptual propositions have been advanced to describe how athletes may coordinate, control, and organize movements under the challenging demands of competitive sporting environments (3–5). Traditional models, derived from cognitive and experimental psychology, have been adopted, primarily taking an organism-centered perspective by emphasizing psychological and neural processes, such as memories, knowledge acquisition, and the processing of information indirectly through representations stored within the brain (3, 6). However, our conceptual analysis highlights an *ecological dynamics* framework to problem-solving within sport, steeped in a systems orientation, addressing the ongoing relations between oneself and the environment (3). Utilizing an ecological perspective, we will aim to unpack how the movement behavior that emerges in sport could be viewed as a problem-solving activity for the athlete, where the integrated processes of perception, cognition, and action underpin the movement solutions coupled to performance problems through relevant information sources in the environment. This distinction could offer readers a greater perspective to more comprehensively narrate the movement problem-solving story within sport, by respecting the individual (with unique “effectivities” or characteristics), the performance context (i.e., the problem), and the content (i.e., the specific movement solution to emerge).

## Problem-solving in the context of sport

According to the Merriam-Webster Dictionary, the definition of *problem-solving* is simply, “the process or act of finding a solution to a problem.” Other resources may mention the idea of complexity and/or difficulty as it pertains to specific challenges to be addressed in solving a problem. Across many contexts, the use of the term problem-solving, or contemplation around the emergence of a solution, often conjures up an image of a mostly mental construct or a process that is neuro-cognitively driven (3, 7, 8). However, when investigating the (re)organization of *movement solutions* in skill adaptation (8), especially in the challenging athletic contexts of sport performance, the problem-solving process and the movement outcome as a solution, may differ from other domains. We reject problem-solving as proposed by (captured within) traditional computational theories (e.g., information-processing; 9, 10), as these assume an *organismic asymmetry* (11), suggesting that individuals only have “indirect” access to the information in the world that needs processing (interpretation), and movement actions come to be due to stored representations within the brain (8, 12).

In this paper, we discuss ecological ideas on learning to solve movement problems when interacting with a dynamic performance environment, which is contextually dependent on a rich mix of actions, perceptions, cognitions, and knowledge of the environment, drawing on the insights of Gibson (13), Bernstein (2), Newell (14), Kugler and Turvey (15). When a theoretician, researcher, or coaching practitioner utilizing an ecological dynamics framework, is referring to problem-solving as it pertains to the context of an athlete moving skillfully within sport, they are not referring simply to content knowledge about the environment as the sole basis of information underpinning their perceptions, cognitions and actions (16). So, what exactly are they attempting to explain?

The goal of the problem-solving process in sport is oriented around the organization of a solution that adequately addresses the most pertinent issue(s) confronting the athlete (i.e., the problem solver) at that point in time. The emergence of the most effective solution will be largely dependent on the contextual situation since the athlete is typically engaged in some form of motion when interacting with the dynamics of the competitive environment. The interactive *problem-solution dynamics*, demanding changing states of organization to adapt to contexts of sport competition environments, differ significantly from that which is typically studied, understood, or explained across behavioral contexts where long-term memory or information processing dominates. Instead of just relying on knowledge about the environment stored in long-term memory, athletes are required to interact with information that is continually emerging, changing, and unpredictable, relevant to organically resolving an *alive movement problem* (17), which will differ (subtly or significantly) each time it is faced. At a certain level, no two problems in sport are ever truly the same; thus, no two solutions will be the same either. Similarly, two significantly different solutions could emerge to solve the issues or challenges present under the constraints of what appear to be similar problems (4).

The movement problem-solving process in sport often takes place under considerably different constraints than problems presented in other domains. An athlete rarely has the luxury of identifying, or deliberating on, a list of alternative options to employ in trying to solve a problem. There simply is not the time, nor the need, to thoroughly analyze the problem to find out its causes in the way one might in other settings. Many sports have problems that change from moment to moment, presenting a variety of opportunities to solve them in authentic, yet functional, ways (18, 19). Navigating movement problems in this inherently complex and often challenging environment requires athletes to detect and use highly specifying information to make decisions and organize functional movement solutions (20, 21). Gibson (13) spent a long time conceptualizing the nature of the information used to regulate interactions with the environment, where he argued that deeply relevant information resided in the structure of the surrounding environment. Namely, the structure of the surrounding energy arrays may provide information that specifies *affordances* (13)—how an individual can interact with surfaces, objects, other people, and events, for example, especially when in motion. The concept of affordances (which we will expand on later) describes the action-relevant properties of the environment: the opportunities or possibilities for action within the solving of a movement problem (13). Affordances point both ways, referring to both the environment and the animal in a way that no other term really does—linking the athlete and the environment, as well as the problems and the solutions (13), making them inseparable in both our studies and explanations.

An ecological approach emphasizes the value of *knowledge of* the surrounding structures of the environment in interactions more than the content *knowledge about* the environment (8, 13, 22). Our conceptual analysis maintains distinctions between a coach's over-reliance on knowledge about the performance environment which defines instructions, descriptions and feedback to instruct athletes during learning, prominent in traditional pedagogical methods. An emphasis *on* knowledge of the performance environment underpins actual behavioral interactions (involving perception, cognition, and action), from an ecological perspective. We draw attention to the importance of designing learning environments to enhance athlete-environment interactions (rather than instructing players what to think, perceive and do—the traditional behaviorist perspective). Through using knowledge of the performance environment, athletes can learn to utilize affordances available in manipulated task constraints and through coupling their perception and action, framed through intentionality. This conceptualization has been advocated in ecological dynamics and was recently summarized in the work of Woods and Davids (23) which highlighted potentialities of coaches facilitating athlete learning through “making/doing and not telling”.

### Skill according to an ecological dynamics framework

Based on these insights, from an ecological dynamics perspective, Araújo and Davids (8) reconceptualized *skill acquisition* as “the emergence of an adaptive, functional relationship between an organism and its environment.” Skillful movement behaviors, therefore, when viewed through an ecological lens, could be conceived of as the emergence (with practice and experience) of coordinated movement solutions by an athlete, which are essentially dynamical products of continuous performer-environment interactions. This ecological view moves skill acquisition away from acquiring content knowledge about the environment towards skill in adapting to the dynamics of the environment (and its problems) and begins to position the expression of skill closely to that of effective problem-solving through one's movement.

Though many associate the work of Nikolai Bernstein with ideas of coordination in movement systems (i.e., *Bernstein's degrees of freedom problem*), it is clear Bernstein felt that a more appropriate scale of analysis for movement should extend beyond the motor system, looking deeper into relations and interactions between systems—connecting movement problems with the solutions coordinated to solve them. According to Bernstein (24), “dexterity is the ability to find a motor solution for any external situation, that is, to adequately solve any emerging motor problem.” He added, “dexterity is not confined within the movements or actions themselves but is revealed in how these movements behave in their interaction with the environment, with its unexpectedness and surprises”. The key to skill adaptation is the emergence of movement solutions, softly assembled (15) to functionally fit the unique and unfolding problem.

The aforementioned work (2, 13, 22, 24) helps shape the systems narrative around the tight coupling between perception and action and the athlete and their environment (i.e., signifying the fit between problem and solution). Viewed in this way, animals and the surrounding environment are inseparable and incapable of existing without one another (13), as they have a mutual and reciprocal relationship with each other; this idea underlines the relations between athletes and their movement problem-solving processes within competitive performance. Athletes cannot organize a solution without first being presented with a problem. This notion implies that scientists should not analyze or attempt to understand movement solutions in isolation without paying equal respect to the contextual problem the behavior is organized to satisfy.

If the essence of movement behavior in sport is in the problem-solving activities which are dynamically and continuously emerging from the athlete-environment relationship, then the questions arise: How does an athlete coordinate the proper relations of their movement system with the environment? Furthermore, what individual subsystems, and associated processes, may underpin this movement problem-solving process?

## The integrated movement problem-solving process

To understand the phenomenon of emergent movement behavior in sport, Seifert et al. (19) acknowledged that: (a) it occurs at the ecological scale of analysis (i.e., studied through the ongoing, reciprocal relations between the athlete and the environment); (b) perception and action are viewed as emerging from these interactions; and (c) it is predicated on the circular causality of the relationship. Under an ecological framework, the movement behaviors in sport, reconceptualized as problem-solving activities carried out by the movement system of the athlete, are subserved by the coordination of perception and action with respect to information in the world.

### Intertwined nature of perception, cognition, and action

The above contention captures the importance of individuals—in our case, athletes—being both *perceivers* and *behavers*, where perception and action processes can be thought of as tightly coupled because perception is required to adequately regulate behavioral actions and acting allows for the pick-up of additional information about the problems in the world, which will further serve to guide subsequent actions in a tightly coupled fashion (13, 25). Within this, the concept of *behavioral dynamics* (26), which integrates an information-based approach to perception with a dynamical systems approach to the organization of action (27), may enable further understanding as to how detected information in person-environment relations channels the movements which emerge in the pursuit of goal-directed actions. At a certain level, the problem of the organization of behavior is synonymous with perception and action processes feeding into one another as movement becomes coupled to information (27).

**Figure 1:**
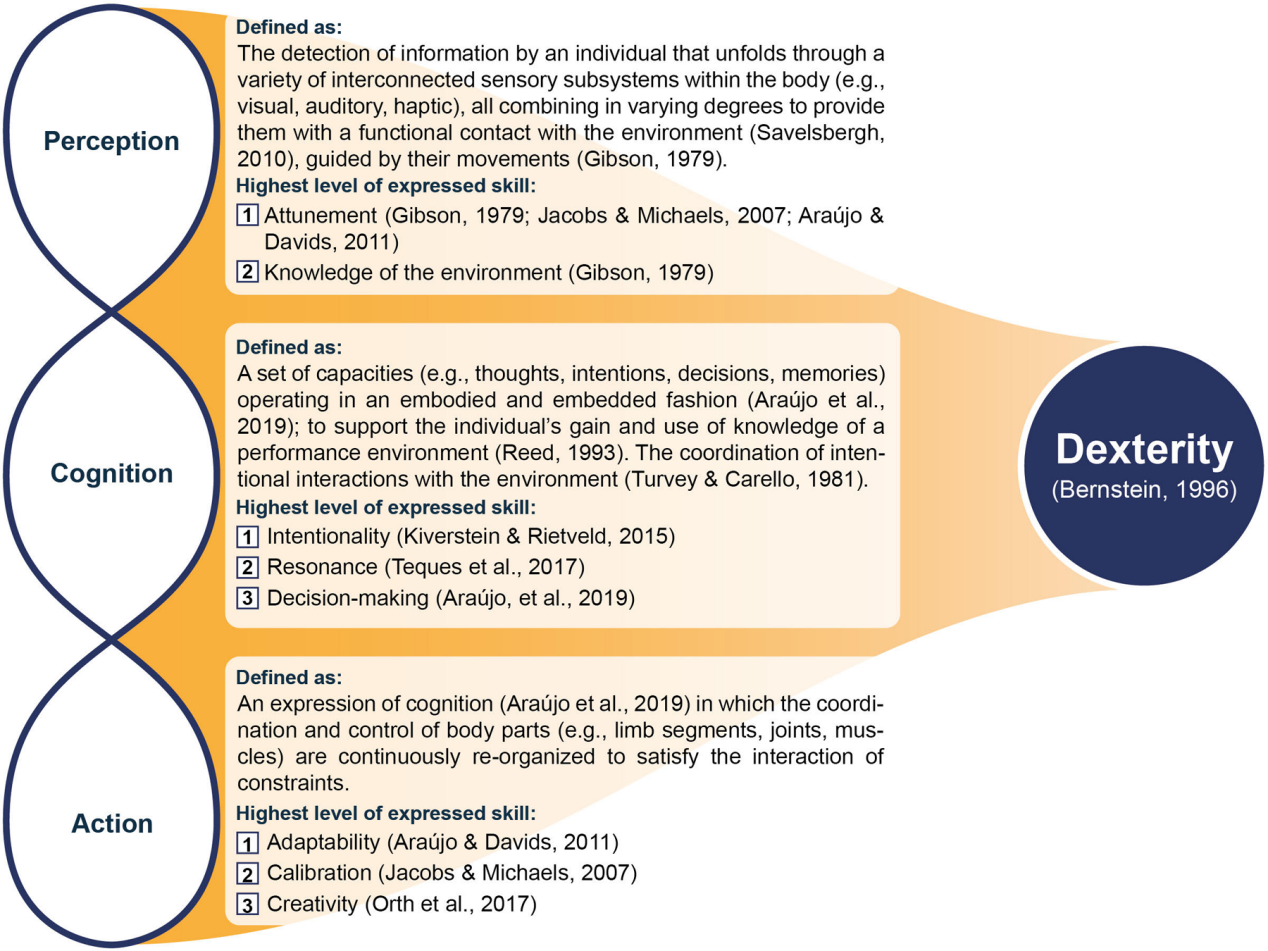
Processes of the human movement system. Defining the interwoven processes of the human movement system and their interdependent contributions to carrying out movement behaviors in sport within an ecological dynamics framework, on the path towards the expression of dexterity.

The current state of affairs between the unfolding problem-solution dynamics can be assessed through perception of information (see Figure 1) which is a dynamic process involving the entire human movement system of the athlete (16, 28). With increased exposure to practice and performance, athletes may become more sensitive to (i.e., *perceptually attuned to*) which informational variables to attend to and when to attend to them. Practice and experience should *educate their attention* to these variables, allowing athletes to more functionally regulate their movement actions across various situations (8, 29). Through this process of attunement, the athlete can create constant, purposeful contact with a complex, ever-changing environment, through the perception of information and a range of decisions that they actively undertake, regulating their interactions to solve emergent problems.

It should also be acknowledged that emerging movement solutions in sport are always goal-directed. Meaning, movement solutions are organized for the functional purpose of achieving a specific outcome in the competitive performance environment. Therefore, performance behaviors need to be interpreted with reference to that functional purpose (26, 30). Accounts of movement behavior in sport must capture the nature of the athlete's intended goal(s), seeking to characterize how movement solutions are coordinated in relation to the problems and challenges of the environment. This could point to a further understanding of how cognitive processes may be situated in the emergence of movement behavior (6, 31). Though there have been questions raised over a lack of explanation about how cognition may function in ecological theories (3, 5), perception could be considered a fundamental type of cognition (13). Additionally, cognition may also be considered the coordination of intended interactions with the performance environment (32). Finally, Reed (33) called cognition “a set of capacities by which observers gain knowledge of their environment”. Cognition, provided by perception, could support an athlete's knowledge of the environment, allowing them to be aware of events, objects, and others that exist (attention), have existed (memories), may come to exist (anticipation), and ought to exist (planning, prediction). According to Reed (33), actions, perception, and cognition are “knowledge-yielding processes”. Perception is a cognitive function of the most essential kind because it yields knowledge of affordances available in a performance environment.

Therefore, an athlete's cognitions may scaffold the information they perceive while also influencing how they ultimately coordinate their actions (20, 34). In an ecological approach, cognition is proposed to operate in an embodied and embedded fashion (6). Here, cognitive processes combine with other subsystems to form a comprehensive, integrated system, supporting the use of knowledge of a performance environment. This unfolds potentially through a *resonance mechanism* in the central nervous system where an athlete becomes tuned to available information (28). For example, if a basketball player perceptually picks up a teammate entering the paint near the basket, the ball handler needs to be “tuned in” to the available information in the performance environment for an affordance (e.g., a passing lane opening up to throw the ball to a moving teammate who is separating from a defending opponent) that invites a quick pass from behind the three-point line. Following Gibson's ideas, when the teammate is detected near the opponent's basket, the point guard's perceptual system resonates with that information for an affordance (13, 28). Further, this has the potential to give rise to a mover displaying *skilled intentionality* by being open and responsive within a rich landscape of opportunities (30, 35), where the affordance landscape will then channel the specific way the ball handler acts (e.g., when, where, and how to coordinate motor system degrees of freedom to complete an accurate and timely pass to the teammate).

Though we may be able to neatly define each of the various processes (see Figure 1), because of their tightly intertwined nature, perception, cognition, and action would not be viewed as separate processes, functioning in isolation, under an ecological dynamics framework. Instead, perception, cognition, and action could be considered inextricably linked as the constituent, interacting elements of one *integrated movement solution* (IMS)—in slightly differing, yet highly related, accounts of a holistic, *movement problem-solving process* (MPSP).

### Continuous (re)organization of system degrees of freedom

**Figure 2:**
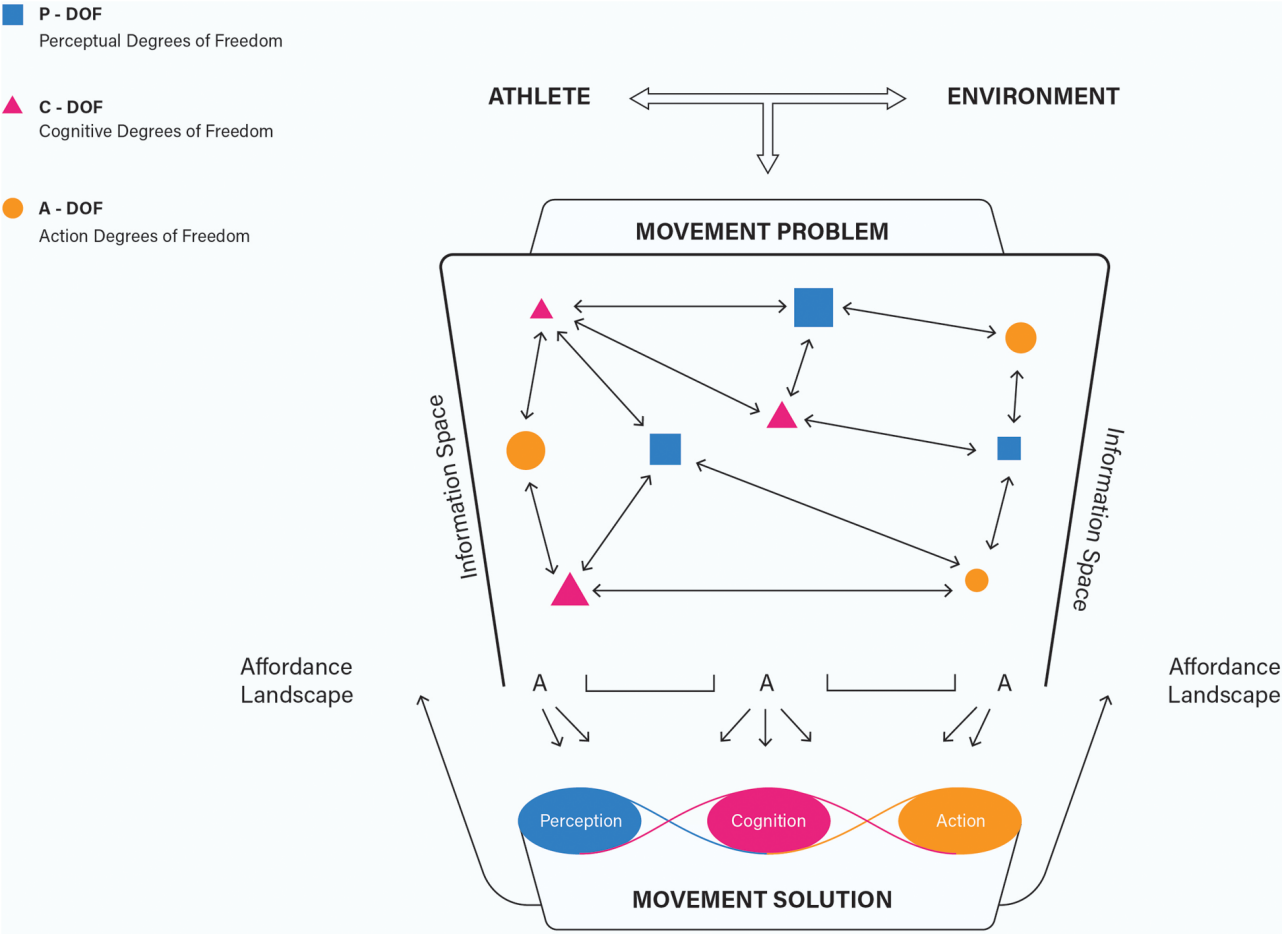
The integrated movement problem-solving process. The integrated nature of the processes of perception, cognition, and action underpinning the Movement Problem-Solving Process (MPSP) as an athlete coordinates system degrees of freedom in relation to specifying information and affordances present in solving a movement problem.

Context requires an individual to adapt to changing external demands (i.e., constraints where a movement emerges) and internal states (i.e., within the individual). Newell (14) proposed that *coordination* could be defined as the function constraining the free variables of the human movement system (i.e., degrees of freedom) into a behavioral unit—capturing how the component parts and processes of a system come into relation with one another. What an athlete is attempting to coordinate is not just a motor response, but a *functional behavioral unit*, organized to fit the dynamical needs of their world. We propose that the IMS is a functional behavioral unit that harnesses degrees of freedom within and between the dimensional levels across the system (of perception, cognition, and action) and points us to how those processes may be integrated to underpin this MPSP (see Figure 2).

Ultimately, this problem-solver paradigm extends Bernstein's degrees of freedom problem beyond the motor system, looking deeper into the relations and interactions between subsystems and processes. Just as there are numerous ways that the degenerate motor system can be coordinated and controlled (e.g., joint positions, timing and sequencing of patterns), there will be degrees of freedom perceptually and cognitively as well. For example, perception can unfold through a variety of interconnected sensory subsystems within the body (e.g., visual, auditory, haptic), all combining to varying degrees to provide the athlete with functional contact with the world (36), as they attempt to detect the most relevant information regarding opportunities present for authentically solving a peculiar problem. Closely connected to perception, the athlete's cognitive degrees of freedom can also be coordinated in various ways, led by their constantly changing intentional aims, the diverse thoughts they may have, their unfolding movement strategies, and the wide range of decisions that they can make as a mover interacting with the dynamic problems within the world.

Processes of perception, cognition, and action are interdependent with one another, interacting with circular causality, underpinning movement systems as complex, dynamical systems (20). Through these interconnections, dynamics in the organization of the self-regulation process at a local level (such as the specifying information a player is picking up, how and when the athlete is picking up the information, etc.), could perpetually feed into the dynamics of other processes (e.g., intentions underpinning performance, movement strategy employed, how actions are being regulated), as well as global, system-wide movement behavior. Skill adaptation is enhanced because constituent parts and related processes of a human movement system can fit together and operate in many different ways or configurations (i.e., displaying system *degeneracy*; 37).

The IMS, or the MPSP, can be investigated at and across multiple timescales of analysis, ranging from the local interactions of movement performance (i.e., brief moments in time or numerous sequences unfolding successively in a singular play) to more global patterns of evolved movement behavior indicative of skill acquisition and learning over significant periods (i.e., days, weeks, months, or years). In sports, movement problem-solving may not be adequately captured through the expression of a discrete event, isolated entity, or simplified response, as it is occasionally reduced to within traditional motor control or learning research (e.g., button-pressing, stimuli-response aiming tasks). Within the typical performance constraints of most sports, we may not always be able to accurately determine where one movement solution, or even one movement problem, ends and another one begins for the athlete. Due to context-conditioned coupling variability (38), one movement problem does not typically exist in isolation because it is integrated with other deeply interrelated, interconnected challenges and problems. Thus, the MPSP could be best conceptualized as a constantly unfolding process where the system's degrees of freedom are continuously (re)organized, and circular causality is displayed throughout the relations between system components (see Figure 2, which shows arrows pointing both ways between the degrees of freedom of each of the human movement system's dimensional levels to depict the potential relationships emerging during movement behavior organization). To illustrate, imagine a soccer player dribbling a ball, concurrently scanning the landscape of the performance environment for opportunities to pass to a teammate, execute a shot on goal, or continue to move throughout space, navigating around defenders and teammates in an evasive manner. Moment-by-moment, various local movement problems, challenges, and decisions will continue to emerge and unfold dynamically, which the player must attempt to deal with in their own unique and authentic fashion. These problem-solution dynamics could be studied as such on any number of micro or macro levels.

Recent research (see 4, 17, 20, 21, 39) has extended our understanding of these theoretical constructs in ecological dynamics, elaborating how these ideas can be practically utilized in skill enhancement programs. In particular, there has been a rhetoric threaded throughout this work on the need for athletes to seek functional movement solutions and adaptive behaviors. For example, when presenting their idea of *wayfinding*, Woods et al. (39) highlighted how movers purposefully and skillfully regulate through their environment underpinned by this deeply entangled relationship of perception, cognition, and action; encompassing real-time, actively engaged problem-solving.

## The concept of affordances

As outlined, to sufficiently understand movement behavior in sports, one should investigate the problem-solution dynamics at hand, and the reciprocal nature of their changing states of co-organization. To assist us in comprehensively doing so, it could be helpful to utilize the concept of affordances. Acknowledging affordances as an emerging relationship between an individual and the environment (13, 40) and therefore creating a link between the two, as well as the problems and the solutions, has clear implications that become relevant to problem-solving in sports. Because it is the information within the world which specifies the affordances offered to an athlete, affordances bring a needed practical perspective on the role and use of information in the solving of movement problems (16).

### Affordances as functional semantics

Affordances have been positioned as a conceptual pillar for the study of movement behavior within sports (41). In particular, Fajen et al. present the key features of affordances that allow them to be especially helpful in investigations. They highlighted how affordances (a) are real; (b) are animal-specific; (c) capture the reciprocity and coupling of perception-action; (d) allow for prospective control; (e) are meaningful; and (f) are dynamic. Due to their ability to provide the athlete with information about their ongoing relationship with the world, informing individuals how they could move to achieve their behavioral goals (i.e., solving problems in the performance environment), affordances could be viewed as *functional semantics* for sports (41).

Affordances go beyond being just mere possibilities or opportunities for acting in a certain way within a performance environment as they could also be viewed as invitations or solicitations (34); it is almost as if the environment is calling for a specific way of acting, connecting the performer-environment through the constant exchange of information. At any moment, there will be a variety of simultaneous and successive affordances which are available for detection, each inviting or soliciting a player to act, though they will differ in degree of attraction for various reasons. Thus, the athlete will still need to decide which affordance to select and act upon after perceiving them out of a rich landscape of inviting opportunities. Ultimately, agency is needed in accepting affordances as the athlete will still be the source of their own activity and the vehicle of the emerging movement behavior.

In recent work by Araújo and colleagues (6), more elaborate hypotheses were offered to depict how cognitive processes, particularly those related to decision-making, may exist as constituent elements of the IMS that emerge as one perceives, selects, and acts upon the affordances in the world. In their work (see 31, 42), decision-making is closely linked to problem-solving. Because *the world is its best model* and behavior is always dependent on circumstances, both cognition and decisions will also be emergent processes, like perception and action (6, 20). Generally speaking, perception is of affordances, and actions, which are an expression of cognition, are the realization of the selected affordances (6).

### Information and affordances within sport contexts

As noted earlier, the potential affordances connecting the athlete-environment system are specified by information provided by surrounding energy arrays, residing in the structure of the sporting environment. Thus, the movement solutions that emerge will depend on an athlete's sensitive detection of and close coupling to these relevant information sources. Though additional research is certainly needed, some research has begun to explore the nature, role, and use of information in the regulation of movement within some sport contexts (e.g., 16, 41), such as team sports like rugby codes (42–44) or basketball (45), as well as various combat sports (46, 47).

Taking this research into account, it would seem that information pertaining to interpersonal distance (46, 47), particularly within dyadic relationships (e.g., between opponents or teammates), could be an informational variable specifying affordances, channeling the movement problem-solving processes of athletes. As Gibson (13) stated, “Behavior affords behavior,” meaning it is how two (or more) individuals interact in the competitive performance environment that gives rise to the possibilities for athlete performance behaviors to emerge. Though the informational variable of interpersonal distance may be transferable across some sports, its critical values (i.e., how this information will be utilized in the regulation of movement) will be sport-, context-, and individual-specific. Additionally, it is likely that information about interpersonal distance alone may not be enough to explain the movement behavior that emerges in context (43). In other words, athletes may also be attuning to other informational variables to specify the affordances present when faced with solving complex movement problems. These variables could relate to relative angles or velocity values between competitors (42, 44), variations in gaps between opponents and teammates (17, 20), postural characteristics of opponents (17, 45), or any combination of these variables (17, 20, 44).

## Discussion

Researchers and practitioners alike may begin to adopt ideas related to the themes presented within this conceptual analysis, ultimately shifting their paradigm to one oriented around movement behavior as a problem-solving activity. When one's scope of analysis and perspective changes in this way, embracing an ecological dynamics framework and problem-solver paradigm, it can lead to tremendous changes in the ways they approach their craft, what they attempt to study in their investigations, and how they may set-up their environments in order to capture (i.e., scientists) or facilitate (i.e., coaches) the emergence of skill.

### Implications for research

In contemplating where the fields of movement, learning, and pedagogy could advance to better understand how movement solutions emerge in different performance contexts of sport, it is worth highlighting some additional areas for future research to consider. First, there is an apparent paucity of research that studies sport movement skills *in situ*. Though it is obviously a daunting and possibly intrusive task to adequately unpack movement behavior in this fashion (i.e., in competitive performance contexts), we must recognize the need and importance of this endeavor. In studying movement behavior: context is everything! The problems which confront athletes, as well as the coordination of IMS which emerge, will be highly specific to the performance context which they inhabit (48).

Second, it has been well over a decade since Fajen and colleagues proclaimed the potential for the use of affordances in studying the problem-solution dynamics in sports (41). Yet, there is still limited research that has attempted to fully explain the presence of affordances across a variety of sporting contexts, while addressing their explanatory role in how they may influence the emergence of movement behavior. There is a need for research that can further unpack the MPSP in terms of the intertwined processes of perception, cognition, and action in relation to the opportunities detected, selected, and acted upon out of the rich landscape of affordances (6).

The adoption of this problem-solver paradigm (underpinned by ecological dynamics) is useful for enhancing our understanding of the problem-solution dynamics in sports that are beyond invasion-based team games (e.g., soccer, rugby codes, basketball, American football) or dyadic relationships in combat sports, as we explored earlier. Alive movement problems are everywhere and within all sporting environments, and they require athletes to organize an adaptable IMS. Researchers should continue to investigate the potential informational variables that performers are detecting, and how they are organizing their movement behaviors in relation to the affordances they perceive, both within and across diverse sporting contexts. Some research has been conducted in this area (see 49–53 for examples), but it needs expansion to further impact practice.

Additionally, many of the investigations into information, affordances, and movement behavior in sport have been conducted with athletes who perform at relatively low levels of skill. To understand the problem-solving processes of athletes across the timescales of learning, we should study how those who display dexterity (i.e., the ability to solve any emerging movement problem) interact with the varied and complex problems of their world, through their use of specifying information, differently from their less skilled counterparts. There is a need to understand more about the behavioral organization of the MPSP of highly skilled, elite athletes and how the degrees of freedom across the dimensional levels of their movement systems (i.e., perception, cognition, and action) are coordinated.

### Practice design for the facilitation of dexterity

Supported by ideas underpinned by theory and empirical evidence, coaches and support practitioners can design practice environments to facilitate enhanced movement problem-solving for the athletes they partner with in training. Information sources that athletes become attuned to, as well as their common ways of behaving, are influenced by the situations and conditions that they routinely face in practice environments. Thus, coaches should aim to set alive movement problems (17) to present to the athlete in practice and training. These types of problems are those where the strategies and outcomes are unpredictable (e.g., the inclusion of moving opposition and teammates in team sports situations, a resisting opponent in combat sports). Here, athletes will be required to perceive a vast array of informational sources specifying a rich landscape of affordances to potentially interact with, actively make decisions around, and coordinate functional actions, often in more variable fashions, in relation to (6, 20). As affordances emerge and decay dynamically within these alive movement problems, it could allow for the search, discovery, and exploitation of a wider range of authentic IMS to positively transfer into perceiving and accepting affordances in the competitive sports arena (20). Yet, many training or practice settings often contain highly sterile and predictable tasks which expect an athlete to simply repeat an idealized motor response with little emergent problem-solving or decision-making needed (i.e., the equivalent of *less alive* movement problems consisting of *dead* technical patterns). To assist coaching practitioners in designing *more alive* movement problems, it may be useful to ask a number of pertinent questions regarding the inclusion of key conceptual principles within activities being presented to athletes in any practice environment (see Table 1).

**Table 1:** Alive movement problem setting checklist.

Does the movement problem presented to the athlete in practice:
1. Keep the problem-solution relationship intact?
2. Present a task disposition representative of the competitive performance environment?
3. Contain relevant sources of information for the athlete to regulate their movements?
4. Keep perceptions, cognitions, and actions deeply intertwined?
5. Maintain a practical and relevant goal as an intention to channel the movement solution?
6. Allow for the continuous (re)organization of system degrees of freedom?
7. Require the athlete to authentically connect to the problem in their own unique way?
8. Maintain a certain level of unpredictability, requiring the athlete to actively make decisions as needed?
9. Present emerging and decaying affordances?
10. Change in some meaningful way each time it is faced (i.e., repetition *without* repetion)?
The more questions you answer “yes” to, the more likely it is that an *integrated movement solution* will emerge that is functionally fit for the peculiar movement problem, guiding the athlete *to become one with* the movement problem.
If you answered “no” to any of the above questions, how can you adjust the movement problem by manipulating relevant constraints to make it more *alive*?

Pertinent questions for coaching practitioners to ask when designing alive movement problems in practice for the athlete's pursuit of organizing functionally fit movement solutions transferable to competition.

Research questions may still exist on: (i) how to ensure representativeness of practice designs (i.e., for the movement problems presented); (ii) how to appropriately set a movement problem that adequately stretches a specific athlete's movement behavioral dynamics to a place of continued evolution; (iii) how to determine if a movement solution is ‘correct’; and (iv) how to effectively guide a performer's perception, cognition, and action processes to become functionally fit to match the problems in their world. However, one thing is clear: dexterity in sport does not equate to repeating a particular movement pattern more consistently or employing a given strategy more automatically (2). Instead, dexterity centers on the athlete being able to solve any emerging movement problem (20, 21, 24), across situations (e.g., facing opponents with different capabilities or styles of movement skill, operating under various spatial and temporal demands), and conditions (e.g., in various weather conditions, facing pressure, under levels of fatigue), allowing them *to become one with* a dynamic, complex problem through interactive information transactions.

For highly interactive problem-solving to occur, the complexion and disposition of most movement problems in practice should vary frequently from repetition to repetition. This type of learning opportunity could allow athletes to gain experience in functionally self-regulating the processes involved with their movement problem-solving—thus aligning with Bernstein's (2) original thoughts on *repetition without repetition*—with the goal of the mover becoming skillfully attuned, intentional, and adaptable. Woods and colleagues (39) describe this process of learning in practice, emphasizing that coaches should “challenge the learner to experiment through performing, adapting and creating movement solutions that best answer his/her individual needs within a given context.” Ultimately, by navigating through the solving of a diverse range of alive problems, athletes will learn how to coordinate their perception, cognition, and action by continuously (re)organizing the degrees of freedom of the human movement system and adjusting the relations between these integrated processes to fit together in various ways. Through this exploration and search process, athletes strengthen their connection to the unique problems of the environment, tightly coupling their movement in close relation to the affordances offered within those problems.

When athletes solve movement problems in this highly emergent fashion, practitioners can expect the expression of a number of signature movement properties^[1]^ (e.g., variability, abundance, creativity, authenticity), sometimes viewed as undesirable in more traditional approaches. In an ecological dynamics framework, these respective movement properties are not to be frowned upon or avoided; instead, they should actually be pursued as they are recognized as key performance indicators of skillful and dexterous movers (19). Recognizing that all athletes have their own individual constraints and movement histories, practitioners may be well served to avoid imposing their own idealized ways of perceiving, deciding, and acting upon the athlete (54). Instead, skill optimization may stem from attunement, abundance, authenticity, and adaptability. Being that no two problems in sport are ever truly the same, and sporting environments can present a diverse range of problems to athletes, it would stand to reason that the athlete having a plethora of potential movement strategies and options that can be flexibly adjusted to meet the needs of a peculiar problem becomes a worthy aspiration for coaches to pursue. Thus, learning environments should also reflect this goal.

To assist in the emergence of highly functional and authentic movement solutions, coaching practitioners, acting as learning environment designers, should aim to guide and facilitate, employing communication methods (i.e., instructions and feedback) that strive to educate the attention (pursuing perceptual attunement) and intentions of the athlete (29). The goal of this type of *direct learning* approach would be to encourage the perceptual search for relevant information sources and the affordances they specify, while allowing the athlete to explore the breadth of their entire movement toolbox. As the athlete gains experience and exposure, they will become more attuned to specifying information sources, being intentional about the goals to be achieved in particular situations, and being adaptable in how their movement is calibrated and coupled to the relevant information within the problems of the sport environment.

## Footnotes

^1^A detailed discussion of this related literature is beyond the scope of this conceptual analysis. Interested readers should refer to the works of 18, 19, 20, and 21.
