# Corrigendum: (Re)conceptualizing movement behavior in sport as a problem-solving activity

**DOI:** 10.3389/fspor.2023.1261067

**Published:** 2023-08-09

**Authors:** Shawn Myszka, Tyler Yearby, Keith Davids

**Affiliations:** ^1^ Emergence, Minneapolis, MN, United States; ^2^ School of Natural, Social and Sport Sciences, University of Gloucestershire, Gloucester, United Kingdom; ^3^ Sport & Physical Activity Research Centre, Sheffield Hallam University, Sheffield, United Kingdom

**Keywords:** movement behavior, problem-solving, ecological dynamics, dexterity, perception-action coupling, affordances (ecological psychology)

A Corrigendum on (Re)conceptualizing movement behavior in sport as a problem-solving activity Myszka S, Yearby T and Davids K. (2023) Front. Sports Act. Living 5:1130131. doi: 10.3389/fspor.2023.1130131

**Incorrect Reference**

In the published article, the reference for Araújo et al. (2019) was incorrectly written as “Araújo D, Hristovski R, Seifert L, Carvalho J, Davids K. Ecological cognition: expert decision-making behaviour in sport. *Int Rev Sport Exerc Physiol.* (2019) 12(1):1–25. doi: 10.52547/joeppa.12.1.1”

It should be “Araújo D, Hristovski R, Seifert L, Carvalho J, Davids K. Ecological cognition: expert decision-making behaviour in sport. *Int Rev Sport Exerc Psychol.* (2019) 12(1):1–25. https://doi.org/10.1080/1750984X.2017.1349826”

**Author Name Misspelled in Reference**

In the published article, the name of one of the authors was incorrectly spelled in the reference for “Michaels C, Carello C. *Direct perception*. Englewood Cliffs, NJ: Prentice-Hall (1981).”

It should be “Michaels CF, Carello C. *Direct perception*. Englewood Cliffs, NJ: Prentice-Hall (1981).”

**Author Name Misspelled in Reference**

In the published article, the name of one of the authors was incorrectly spelled in the reference for “Newell K. Change in movement and skill: learning, retention, and transfer. In: Latash ML, Turvey MT, editors. *Dexterity and its development*. Mahwah, NJ: Lawrence Erlbaum Associate (1996). p. 393–429.”

It should be “Newell KM. Change in movement and skill: learning, retention, and transfer. In: Latash ML, Turvey MT, editors. *Dexterity and its development*. Mahwah, NJ: Lawrence Erlbaum Associates (1996). p. 393–429.”

**Incorrect Reference**

In the published article, the reference for Kugler PN, Turvey MT was incorrectly written as “Kugler PN, Turvey MT. *Information, natural law, and the self-assembly of rhythmic movement*. London, UK: Erlbaum (1987).”

It should be “Kugler PN, Turvey MT. *Information, natural law, and the self-assembly of rhythmic movement*. Hillsdale, NJ: Lawrence Erlbaum Associates (1987).”

**Author Name Misspelled in Reference**

In the published article, the name of one of the authors was incorrectly spelled in the reference for “Woods C, Davids K. “You look at an ocean; I see the rips, hear the waves, and feel the currents”: dwelling and the growth of enskiled inhabitant knowledge. *Ecol Psychol*. (2021) 33(3–4):279–96. doi: 10.1080/10407413.2021.1965481”

It should be “Woods CT, Davids K. “You look at an ocean; I see the rips, hear the waves, and feel the currents”: dwelling and the growth of enskiled inhabitant knowledge. *Ecol Psychol*. (2021) 33(3–4):279–96. doi: 10.1080/10407413.2021.1965481”

**Author Name Misspelled in Reference**

In the published article, the name of one of the authors was incorrectly spelled in the reference for “Bootsma R. Ecological movement principles and how much information matters. In: Post AA, Pijpers JR, Bosch P, Boschker MSJ, editors. *Models in human movement science: Proceedings in the second symposium of the institute for fundamental and clinical human movement sciences*. Enschede: PrintPartners Ipskamp (1998). p. 51–63.”

It should be “Bootsma RJ. Ecological movement principles and how much information matters. In: Post AA, Pijpers JR, Bosch P, Boschker MSJ, editors. *Models in human movement science: Proceedings in the second symposium of the institute for fundamental and clinical human movement sciences*. Enschede: PrintPartners Ipskamp (1998). p. 51–63.”

**Author Name Misspelled in Reference**

In the published article, the name of one of the authors was incorrectly spelled in the reference for “Warren W. The dynamics of perception and action. *Psychol Rev.* (2006) 113(2):358–89. doi: 10.1037/0033-295X.113.2.358.”

It should be “Warren WH. The dynamics of perception and action. *Psychol Rev.* (2006) 113(2):358–89. doi: 10.1037/0033-295X.113.2.358.”

**Incorrect Reference**

In the published article, the reference for Teques et al. (2017) was incorrectly written as “Teques P, Araújo D, Seifert L, del Campo V, Davids K. The resonant system: linking brain-body-environment in sport performance. *Progress Brain Res*. (2017) 234:33–52. doi: 10.1016/bs.pbr.2017.06.001”

It should be “Teques P, Araújo D, Seifert L, del Campo VL, Davids K. The resonant system: linking brain-body-environment in sport performance. In: Wilson MR, Walsh V, Parkin B, editors. *Progress Brain Res.* Amsterdam, North Holland: Elsevier (2017) 234:33–52. doi: 10.1016/bs.pbr.2017.06.001”

**Author Name Misspelled in Reference**

In the published article, the name of one of the authors was incorrectly spelled in the reference for “Jacobs D, Michaels C. Direct learning. *Ecol Psychol*. (2007) 19(4):321–49. doi: 10.1080/10407410701432337”

It should be “Jacobs DM, Michaels CF. Direct learning. *Ecol Psychol*. (2007) 19(4):321–49. doi: 10.1080/10407410701432337”

**Author Name Misspelled in Reference**

In the published article, the name of one of the authors was incorrectly spelled in the reference for “Withagen R, Michaels C. On ecological conceptualizations of perceptual systems and action systems. *Theory Psychol*. (2005) 15(5):603–20. doi: 10.1177/0959354305057265”

It should be “Withagen R, Michaels CF. On ecological conceptualizations of perceptual systems and action systems. *Theory Psychol*. (2005) 15(5):603–20. doi: 10.1177/0959354305057265”

**Author Name Misspelled in Reference**

In the published article, the name of one of the authors was incorrectly spelled in the reference for “Turvey M, Carello C. Cognition: the view from ecological realism. *Cognition*. (1981) 10(1–3):313–21. doi: 10.1016/0010-0277(81)90063-9”

It should be “Turvey MT, Carello C. Cognition: the view from ecological realism. *Cognition*. (1981) 10(1–3):313–21. doi: 10.1016/0010-0277(81)90063-9”

**Incorrect Reference**

In the published article, the reference for Reed ES (1993) was incorrectly written as “Reed ES. The intention to use a specific affordance: a framework for psychology. In: Wozniak R, Fischer K, editors. *Development in context: Acting and thinking in specific environments*. Hillsdale, NJ: Lawrence Erlbaum Associates (1993). p. 45–75.”

It should be “Reed ES. The intention to use a specific affordance: a conceptual framework for psychology. In: Wozniak RH, Fischer KW, editors. *Development in context: Acting and thinking in specific environments*. Hillsdale, NJ: Lawrence Erlbaum Associates (1993). p. 45–76.”

**Incorrect Reference**

In the published article, the reference for Withagen et al. (2017) was incorrectly written as “Withagen R, Araújo D, de Poel HJ. Inviting affordances and agency. *New Ideas Psychol.* (2017) 45:11–8. doi: 10.1016/j.newideapsych.2016.12.002”

It should be “Withagen R, Araújo D, de Poel HJ. Inviting affordances and agency. *New Ideas in Psychol.* (2017) 45:11–18. doi: 10.1016/j.newideapsych.2016.12.002”

**Incorrect Reference**

In the published article, the reference for Kugler et al. (1982) was incorrectly written as “Kugler PN, Kelso JAS, Turvey MT. On the control and coordination of naturally developing systems. In: Kelso JAS, Clark JE, editors. *The development of movement coordination and control*. New York: Wiley (1982). p. 5–78.”

It should be “Kugler PN, Kelso JAS, Turvey MT. On the control and coordination of naturally developing systems. In: Kelso JAS, Clark JE, editors. *The development of movement control and coordination*. New York: Wiley (1982). p. 5–78.”

**Author Name Misspelled in Reference**

In the published article, the name of one of the authors was incorrectly spelled in the reference for “Woods CR, Robertson SJ, Davids K. Wayfinding: how ecological perspectives of navigating dynamic environments can enrich our understanding of the learner and the learning process in sport. *Sports Med*. (2020) 6:51. doi: 10.1186/s40798-020-00280-9”

It should be “Woods CT, Rudd J, Robertson S, Davids K. Wayfinding: how ecological perspectives of navigating dynamic environments can enrich our understanding of the learner and the learning process in sport. *Sports Med - Open* (2020) 6:51. doi: 10.1186/s40798-020-00280-9”

**Incorrect Reference**

In the published article, the reference for Blau and Wagman (2022) was incorrectly written as “Blau JJC, Wagman JB. *Introduction to ecological psychology: A lawful approach to perceiving, acting, and cognizing*. 1st ed. New York, NY: Routledge (2022).”

It should be “Blau JJC, Wagman JB. *Introduction to ecological psychology: A lawful approach to perceiving, acting, and cognizing*. 1st ed. New York, NY: Routledge (2022). https://doi.org/10.4324/9781003145691”

**Author Name Misspelled in Reference**

In the published article, the name of one of the authors was incorrectly spelled in the reference for “Esteves P, Oliveira R, Araújo D. Posture-related affordances guide attacks in basketball. *Psychol Sport Exerc*. (2011) 12(6):639–44. doi: 10.1016/j.psychsport.2011.06.007”

It should be “Esteves PT, de Oliveira RF, Araújo D. Posture-related affordances guide attacks in basketball. *Psychol of Sport Exerc*. (2011) 12(6):639–44. doi: 10.1016/j.psychsport.2011.06.007”

**Incorrect Reference**

In the published article, the reference for Chollet D, Seifert L. (2011) was incorrectly written as “Chollet D, Seifert L. Inter-limb coordination in the four competitive strokes. In: Seifert L, Chollet D, Mujika I, editors. *The world book of swimming: From science to performance*. Hauppauge, New York: Nova Science Publishers (2011). p. 153–72.”

It should be “Chollet D, Seifert L. Inter-limb coordination in the four competitive strokes. In: Seifert L, Chollet D, Mujika I, editors. *World book of swimming: From science to performance*. Hauppauge, New York: Nova Science Publishers (2011). p. 153–72.”

**Author Name Misspelled in Reference**

In the published article, the name of one of the authors was incorrectly spelled in the reference for “Seifert L, Orth D, Boulanger J, Dovgalecs V, Herault R, Davids K. Climbing skill and complexity of climbing wall design: assessment of jerk as a novel indicator of performance fluency. *J Appl Biomech*. (2014) 30(5):619–25. doi: 10.1123/jab.2014-0052”

It should be “Seifert L, Orth D, Boulanger J, Dovgalecs V, Hérault R, Davids K. Climbing skill and complexity of climbing wall design: assessment of jerk as a novel indicator of performance fluency. *J Appl Biomech*. (2014) 30(5):619–25. doi: 10.1123/jab.2014-0052”

**Incorrect Reference**

In the published article, the reference for Ade et al. (2017) was incorrectly written as “Ade D, Seifert L, Gal-Petitfaux N, Pozat G. Artefacts and expertise in sport: an empirical study of ice climbing. *Int J Sport Psychol.* (2017) 48(1):82–94. doi: 10.7352/IJSP.2017.48.082”

It should be “Ade D, Seifert L, Gal-Petitfaux N, Poizat G. Artefacts and expertise in sport: an empirical study of ice climbing. *Int J Sport Psychol.* (2017) 48(1):82–98. doi: 10.7352/IJSP.2017.48.082”

**Text Correction**

In the published article, there was an error. A correction has been made to **The concept of affordances**, *Information and affordances within sport contexts*, paragraph 2. This sentence previously stated:

“As Gibson (13) stated, “behavior affords behavior”. Meaning, it is how two (or more) individuals interact in the competitive performance environment that gives rise to the possibilities for athlete performance behaviors to emerge.”

The corrected sentence appears below:

“As Gibson (13) stated, “Behavior affords behavior,” meaning it is how two (or more) individuals interact in the competitive performance environment that gives rise to the possibilities for athlete performance behaviors to emerge.”

**Text Correction**

In the published article, there was an error. A correction has been made to **Discussion**, *Practice design for the facilitation of dexterity*, paragraph 2. This sentence previously stated:

“Research questions may still exist on: (i) how to ensure representativeness of practice designs (i.e., for the movement problems presented), (ii) how to appropriately set a movement problem that adequately stretches a speciﬁc athlete's movement behavioral dynamics to a place of continued evolution, (iii) how to determine if a movement solution is “correct”, and (iv), how to effectively guide a performer's perception, cognition, and action processes to become functionally ﬁt in matching the problems in their world.”

The corrected sentence appears below:

“Research questions may still exist on: (i) how to ensure representativeness of practice designs (i.e., for the movement problems presented); (ii) how to appropriately set a movement problem that adequately stretches a speciﬁc athlete's movement behavioral dynamics to a place of continued evolution; (iii) how to determine if a movement solution is “correct”; and (iv) how to effectively guide a performer's perception, cognition, and action processes to become functionally ﬁt to match the problems in their world.”

**Text Correction**

In the published article, there was an error. A correction has been made to **Discussion**, *Practice design for the facilitation of dexterity*, paragraph 4, footnote 1. This sentence previously stated:

“A detailed discussion on this related literature is beyond the scope of this conceptual analysis. Interested readers should refer to the work of 14, 15, 25, 2.”

The corrected sentence appears below:

“A detailed discussion of this related literature is beyond the scope of this conceptual analysis. Interested readers should refer to the work of 18, 19, 20, and 21.”

The authors apologize for these errors and state that this does not change the scientific conclusions of the article in any way. The original article has been updated.

